# A Mini-Review of Adverse Lung Transplant Outcomes Associated With Respiratory Viruses

**DOI:** 10.3389/fimmu.2019.02861

**Published:** 2019-12-19

**Authors:** Emily S. Bailey, Juliana N. Zemke, Jessica Y. Choi, Gregory C. Gray

**Affiliations:** ^1^Duke Global Health Institute, Duke University, Durham, NC, United States; ^2^Division of Infectious Diseases and International Health, Duke University School of Medicine, Durham, NC, United States; ^3^Global Health Research Center, Duke-Kunshan University, Kunshan, China; ^4^Emerging Infectious Diseases Program, Duke-NUS Medical School, Singapore, Singapore

**Keywords:** lung transplant, adult, respiratory virus, infection, acute rejection

## Abstract

Due to their overall immunocompromised state, lung transplant recipients (LTRs) are at increased risk for the development of viral respiratory infections compared to the general population. Such respiratory infections often lead to poor transplant outcomes. We performed a systematic review of the last 30 years of medical literature to summarize the impact of specific respiratory viruses on LTRs. After screening 2,150 articles for potential inclusion, 39 manuscripts were chosen for final review. We found evidence for an association of respiratory viruses including respiratory syncytial virus (RSV), parainfluenza virus, and influenza viruses with increased morbidity following transplant. Through the literature search, we also documented associations of RSV and adenovirus infections with increased mortality among LTRs. We posit that the medical literature supports aggressive surveillance for respiratory viruses among this population.

## Introduction

Lung transplant recipients (LTRs) are susceptible to a multitude of respiratory tract infections. Viral pathogens of particular relevance in LTRs include influenza A and B viruses, respiratory syncytial virus (RSV), parainfluenza virus (PIV), human metapneumovirus (HMpV), rhinovirus, coronavirus (CoV), picornavirus (PcV), and adenovirus (1–3). While these viruses may result in asymptomatic colonization or self-limited upper respiratory tract infection, there is greater potential for severe infection among LTRs. The severity of infection is contributed to by the marked immunocompromised state of LTRs alongside impaired respiratory mucociliary clearance in the first months after surgery and the unique scenario of the allograft being directly exposed to the outside environment. Respiratory viral infections have been associated with significant morbidity and mortality in LTRs (2–4), and increasingly, associations have been proposed between respiratory viruses and acute allograft rejection (AR) (5), chronic lung allograft dysfunction (CLAD) including bronchiolitis obliterans syndrome (BOS) (2, 6) and/or decreased survival (4). In an effort to more fully examine the relationship between respiratory viruses and adverse outcomes among lung transplant recipients, we conducted this systematic review. We sought to focus upon the associations of respiratory viruses with (1) AR, (2) CLAD (including BOS), and (3) mortality post-lung transplant.

## Methods

We conducted a literature search, similar to that conducted by Vu et al. in 2010 (7) and identified 23 papers since the previous review. We searched the MEDLINE database from 1 January 1985 to 30 December 2018 using the following key words: “lung transplant recipients or immunocompromised hosts,” and “influenza, PIV, RSV, HMpV, CoV, bocavirus, AdV, and respiratory viruses,” respectively. Selection criteria for papers were as described in Vu et al. (7). Briefly, selected articles included original peer-reviewed papers reporting at minimum three lung transplant cases with a description of virus detection methods, as well as a description of clinical endpoints.

## Results

### Search Results

**Figure 1:**
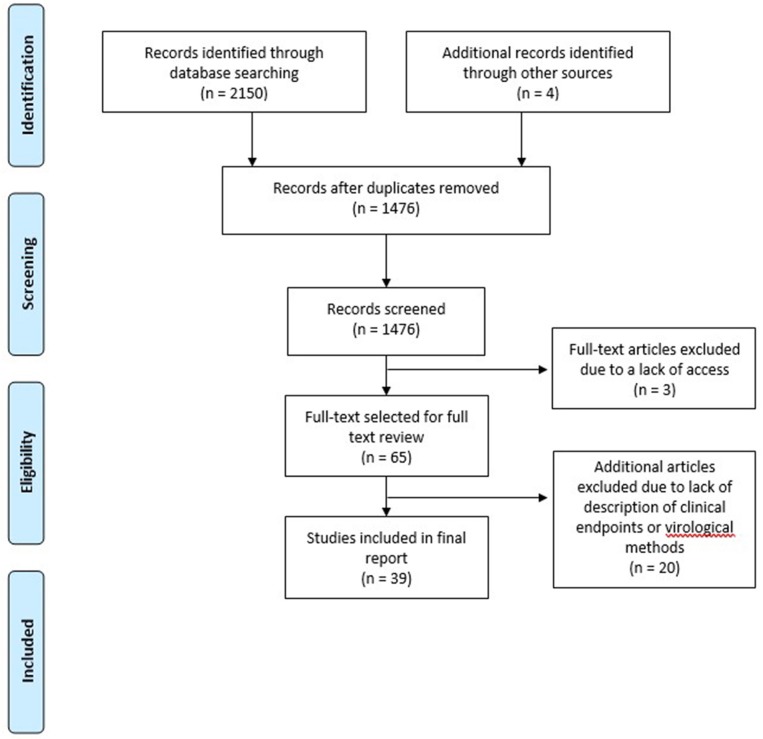
PRISMA flow diagram of the literature search process.

A total of 2,150 articles were identified by our search strategy After duplicates were removed, a total of 1,476 articles were screened for inclusion (Figure 1). Titles, abstracts, and keywords of all English articles were independently reviewed by two authors. Articles that did not describe viral detection methods or clinical endpoints were not considered for full text review. Following the screening of abstracts, 65 articles were selected for full text review. To determine final articles for inclusion, full text review was conducted independently by three authors. In addition to the 65 articles selected for inclusion, an additional four articles were identified from the reviewed articles as potentially significant and these were also reviewed in full. Among these 69 articles, 30 articles were excluded because the virologic detection methods or clinical endpoints were not well-described, resulting in a total of 39 articles being included in this review (Table 1).

**Table 1:** Publications reporting virus association between lung transplant and adverse outcomes.

**References**		**Respiratory virus association with adverse outcomes**		
	**Type of study**	**Acute rejection**	**Chronic lung allograft dysfunction**	**Death**
Kramer et al. (8)	Retrospective	–	AdV, RSV	CMV
Apalsch et al. (9)	Retrospective	–	–	PIV, Flu
Ohori et al. (10)	Retrospective	AdV		AdV
Riise et al. (11)	Prospective Longitudinal	CMV	–	–
Bridges et al. (12)	Prospective	AdV	AdV	AdV
Palmer et al. (13)	Retrospective	–	AdV, RSV	RSV
Vilchez et al. (14)	Retrospective	PIV	PIV	PIV
Vilchez et al. (15)	Retrospective Cohort	PIV, Flu		PIV
Hopkins et al. (16)	Prospective	Flu	Flu	–
McCurdy et al. (17)	Retrospective Case Series	RSV	–	RSV
Khalifa et al. (4)	Retrospective Cohort	RSV, Flu	RSV, PIV, Flu, AdV	RSV, PIV, Flu, AdV
Kumar et al. (6)	Prospective Cohort	Rhino, RSV, Flu, CoV	–	–
Larcher et al. (18)	Prospective Cohort	HMpV	HMpV	HMPV
Sumino et al. (19)	Retrospective Cohort	–	HMpV	–
Humar et al. (20)	Prospective Cohort	AdV	–	–
Kaiser et al. (21)	Prospective	Rhinovirus	Rhinovirus	Rhinovirus
Dare et al. (22)	Prospective Cohort	HMpV	–	–
Hopkins et al. (23)	Prospective Cohort		HMpV, RSV	RSV
Ison et al. (24)	Retrospective Cohort	–	Flu	–
Costa et al. (25)	Prospective Cohort	–	–	Rhinovirus
Engelmann et al. (26)	Prospective Cohort	–	CMV	–
Gerna et al. (27)	Prospective Cohort	–	–	Rhinovirus
Gottlieb et al. (2)	Prospective Cohort	–	HMpV, RSV, Flu, PIV	–
Pelaez et al. (28)	Case Series	RSV	–	–
Bergallo et al. (29)	Prospective Cohort	CMV, Rhinovirus	CMV, Rhinovirus	–
Liu et al. (30)	Retrospective Cohort	RSV, PIV	RSV, PIV	–
Uckay et al. (31)	Prospective Cohort	RSV	RSV	RSV
Weinberg et al. (32)	Prospective Cohort	HMpV, RSV, PIV, Flu	RSV, PIV, Flu, Rhinovirus	–
Ng et al. (33)	Prospective	–	Flu	Flu
Ariza-Heredia et al. (34)	Retrospective Cohort	RSV	–	–
Li et al. (35)	Retrospective Cohort	–	RSV	–
Lo et al. (36)	Retrospective Cohort			AdV
Sayah et al. (37)	Prospective Cohort	Flu, RSV, Rhinovirus	–	–
Bridevaux et al. (38)	Prospective Cohort	Flu, CoV, PcV		PcV, AdV
Schuurmans et al. (39)	Retrospective Cohort	–	Flu	–
Tabarelli et al. (40)	Retrospective Cohort	–	CMV	CMV
Peghin et al. (41)	Prospective Cohort	PcV, CoV, PIV	–	Flu, RSV, Rhinovirus
Liu et al. (42)	Retrospective	–	–	AdV, RSV
Matar et al. (43)	Prospective	–	–	AdV

*AdV, adenovirus; RSV, respiratory syncytial virus; PIV, parainfluenza virus; Flu, influenza; HMpV, human metapneumovirus; CMV, cytomegalovirus; CoV, coronavirus; PcV, picornavirus*.

### Respiratory Viruses and Acute Allograft Rejection

Among the viruses evaluated, RSV was most prevalent and was documented in 9 (23%) of the 39 studies (see Table 1). In a prospective study of community-acquired respiratory viral infections in LTRs, RSV infection occurred in eight (23%) of 35 LTRs and was associated with a high rate of AR (88%) in comparison to other respiratory viruses [HMpV, *n* = 4 (25%); PIV, *n* = 11 (55%); influenza A/B, *n* = 9 (56%); rhinovirus, *n* = 2 (0)] (32). Furthermore, in a retrospective review of 10 LTRs with lower respiratory tract infections with RSV, Uckay et al. found that four patients (40%) had ≥3 AR episodes following laboratory confirmed RSV infection (31). Five other studies also noted single cases of RSV-associated AR in their patient populations (6, 17, 28, 34, 37).

Following RSV, influenza A and B were the respiratory viruses most commonly associated with lung transplant rejection, with seven studies (18%) reporting an association (Table 1). In a retrospective cohort evaluation of LTRs admitted with respiratory viral infections, Vilchez et al. (15) found some degree of AR in 9/15 (64%) of patients diagnosed with influenza respiratory infections (15). Hopkins et al. described a group of nine subjects with influenza that experienced 1.22 episodes of acute rejection on average, compared to 1.33 episodes of acute rejection in a group of nine subjects without influenza infections (23), suggesting that the risk for infection with influenza may not be exacerbated by lung transplant procedures.

PIVs were also identified as an important cause of morbidity, including AR, among lung transplant recipients. In a prospective study of respiratory virus associated morbidity in LTRs, 6/11 (55%) of PIV-infected subjects experienced AR though this was based primarily upon clinical as opposed to histopathologic diagnosis (32). In another prospective study, PIV was detected in 20 lung transplant recipients with histopathologic evidence of AR in 2 (50%) of the four patients undergoing transbronchial biopsy (41). Vilchez et al. documented PIV infection in 24 LTRs (PIV-1 *n* = 7; PIV-2 *n* = 2; PIV-3 *n* = 15) with histopathologic evidence of AR documented in 18 (82%) of the 22 undergoing evaluation (14).

While the data reviewed above supports a possible association between respiratory viruses and AR, there are noted limitations including derivation from retrospective, single center studies with variable definitions of AR and durations of follow-up. Further, conflicting data exists in the literature regarding the association of respiratory viruses with AR in LTRs; for example, Sayah et al. found that LTRs who experienced community acquired respiratory virus infections were not significantly more likely to experience AR than LTRs without infection (37). The relationships of AR and respiratory viruses was assessed in a study examining biopsies from 77 transplant patients, in which Soccal et al. found no association for subjects with AR and respiratory infection (44). Though these authors did not connect specific respiratory viruses with cases of AR, they postulated that respiratory viruses in general may aggravate existing lung impairments and slow recovery, but do not advance AR on their own (44).

### Respiratory Viruses and CLAD/BOS

Similar to the data presented for respiratory viruses and AR, CLAD has been most commonly associated with RSV, influenza viruses, and PIV. In studies examining RSV infected patients, up to 25% of patients experienced CLAD (32), and among patients who received treatment for RSV, many did not develop CLAD (4). Hopkins et al. noted previous BOS in six RSV-infected subjects and documented the new onset or progression of BOS in five RSV-infected subjects (23). Additionally, Uckay et al. found that seven of 10 lung transplant recipients developed new or increased BOS after RSV infection (31); while similarly in a prospective study conducted by Li et al., three RSV-positive LTRs demonstrated BOS at the time of RSV infection, and two others developed new or progressive BOS within 6 months of RSV infection (35).

The pattern of influenza infection in LTRs is seasonally related to the strains of influenza virus that are prevalent. In LTRs infected with influenza A virus, studies have noted that up to 40% of patients were diagnosed with BOS (3, 16) and during the 2009 pandemic H1N1 influenza outbreak, nearly 50% of Australian LTRs developed BOS (33). In LTRs with severe CLAD, such as BOS grade 3, patients with influenza A were unable to successfully regain baseline lung functionality (16).

In contrast to the dual infection pattern (infection before and after surgery) seen with RSV infection, among LTRs, the available evidence indicates that PIV infections most often occurred after transplant surgery. Khalifah et al. conducted a retrospective review of medical records from a large medical university and found that four (57%) out of seven subjects with PIV infection experienced CLAD (4). Similarly, two other studies found that ~45% of PIV-infected patients developed CLAD (2, 32). In a 2001 epidemiological study of PIV infections among LTRs with BOS, three of the four PIV serotypes 1 (*n* = 2), 2 (*n* = 1), and 3 (*n* = 4) were implicated (14). PIV infections and associated CLAD have been demonstrated to have year-round incidence (13, 45), indicating the need for continued surveillance among lung transplant recipients.

There is some evidence that respiratory viruses are predictors of the development of CLAD in LTRs. (4, 12). Palmer et al. documented BOS in 50% of lung transplant recipients who survived respiratory viruses (13), indicating that competing risks may be confounding the true relationship of respiratory viruses and CLAD in LTRs Respiratory viruses may be directly impairing the recovery of lung function in LTRs with CLAD (16), contributing to overall morbidity in LTRs.

### Respiratory Viruses and Mortality

Mortality directly associated with respiratory viruses is difficult to determine in medically complex LTRs. However, the viruses identified by this review as most commonly associated with mortality or patient survival were RSV and AdV. In particular, RSV was associated with 33% of all LTR infections in a pediatric population; however, these infections were not associated with mortality or CLAD (42). Similarly for AdV, AdV—associated mortality has been documented in up to 50% of LTRs with ADV pneumonia (12). Despite this, Liu et al. found AdV infection did not statistically predict mortality (42). In consideration of high mortality rates in LTRs, it is not always clear if death is related to the respiratory infection or other complications of transplant surgery, or infection with other viruses (9, 13, 25). Complications related to surgical procedure, concomitant viruses, secondary infections with bacterial and fungal pathogens, etc. also play a role in lung transplant patient survival. However, studies have directly attributed patient mortality to incidence of respiratory viral infections (33, 41), indicating that respiratory viruses may play a role in lung transplant recipient mortality.

## Discussion

In this review, we found considerable evidence that respiratory viruses are associated with adverse outcomes in LTRs. Similar to Vu et al., which also examined the causal link between respiratory virus infection and adverse outcomes in LTRs, we found that the virus most often linked to these outcomes was respiratory syncytial virus (RSV). In the studies where RSV was reported, it was noted that most cases occurred during the winter months when RSV is most prevalent (17).

In the detection of PIVs, which were reported in association with adverse outcomes in eight (21%) of 38 included studies, cases were reported throughout the year; indicating a need for continual respiratory virus surveillance for LTRs (13, 14, 42). Additionally, in studies that linked the detection of respiratory viruses, such as influenza or AdV, to AR it has been postulated that respiratory infections may initiate other risk factors for rejection (4).

This systematic review had a number of limitations. First, as we limited the online review to studies published between 1985 and 2018, the review may have missed some early reports of respiratory virus association with lung transplant surgery. Second, through our search strategy we focused upon studies that contained both a description of virus detection methods, as well as a description of clinical endpoints and we may have missed some important case studies or reports of infection without descriptions comparable to the other studies included in this review. There is also the possibility of false pathology across the studies reviewed and although our study criteria attempted to limit this issue, it is possible our team missed discrepancies between studies. While our review suggests that respiratory virus infection is likely associated with adverse outcomes among LTRs, there is a need for continued evaluation of this relationship to determine what outcomes are most often associated with specific respiratory viruses.

Our findings convey an important message: the detection of respiratory viruses or the development of a clinical respiratory virus infection among LTRs is often associated with adverse outcomes. Hence, we suggest that intensive, year-round surveillance for respiratory viruses among LTRs is warranted due to the severity and frequency of these adverse outcomes. As multiplex molecular respiratory pathogen assays continue to improve and next generation sequencing becomes more widely available and less expensive, researchers, and clinicians may wish to employ these techniques to support respiratory virus detection and treatment.

## Conclusions

Based on our review of 30 years of medical reports we have summarized compelling evidence that a relationship between respiratory viral infections and adverse lung transplant outcomes exists. From this examination, RSV has the greatest impact on LTRs. Additionally, influenza A viruses and PIVs are a major cause of morbidity and mortality among LTRs. Hence, we argue that these observations support intensive, year-round surveillance for respiratory viruses among LTRs.

## Author Contributions

EB, JZ, and JC conducted the literature review and wrote the manuscript. GG conceived of the idea of the review and helped revise the manuscript to add important scientific content. All authors reviewed the final version of the manuscript and agreed to its submission.

### Conflict of Interest

The authors declare that the research was conducted in the absence of any commercial or financial relationships that could be construed as a potential conflict of interest.
